# Functional and Structural Impact of Deleterious Missense Single Nucleotide Polymorphisms in the NR3C1, CYP3A5, and TNF-α Genes: An In Silico Analysis

**DOI:** 10.3390/biom12091307

**Published:** 2022-09-16

**Authors:** Navakanth Raju Ramayanam, Ranjani Manickam, Vijayakumar Thangavel Mahalingam, Khang Wen Goh, Chrismawan Ardianto, Poovi Ganesan, Long Chiau Ming, Rajanandh Muhasaparur Ganesan

**Affiliations:** 1 Department of Pharmacy Practice, SRM College of Pharmacy, SRM Institute of Science and Technology, Kattankulathur 603203, India; 2 SRM-DBT Platform for Advanced Life Science Technologies, SRM Institute of Science and Technology, Kattankulathur 603203, India; 3 Faculty of Data Science and Information Technology, INTI International University, Nilai 71800, Malaysia; 4 Department of Pharmacy Practice, Faculty of Pharmacy, Universitas Airlangga, Surabaya 60115, Indonesia; 5 Department of Pharmaceutics, College of Pharmacy, Mother Theresa Post Graduate and Research Institute of Health Sciences Gorimedu, Puducherry 605006, India; 6 PAP Rashidah Sa’adatul Bolkiah Institute of Health Sciences, Universiti Brunei Darussalam, Gadong BE1410, Brunei; 7 Department of Pharmacy Practice, Sri Ramachandra Faculty of Pharmacy, Sri Ramachandra Institute of Higher Education and Research, Porur, Chennai 600116, India

**Keywords:** glucocorticoid resistance, computational study, pharmacogenomic, precision medicine, missense mutation, SNP

## Abstract

Human diseases are generally influenced by SNPs (single nucleotide polymorphisms). The mutations in amino acid residues generated by deleterious SNPs contribute to the structural and functional diversity of the encoded protein. Tumor necrosis factor-α (TNF-α), Glucocorticoid receptor gene (NR3C1), and Cytochrome P450 3A5 (CYP3A5) play a key role in glucocorticoid resistance susceptibility in humans. Possible causative mutations could be used as therapeutic targets and diagnostic markers for glucocorticoid resistance. This study evaluated the missense SNPs of TNF-α, NR3C1, and CYP3A5 to predict their impact on amino acid changes, protein interaction, and functional stability. The protein sequence of dbSNP was obtained and used online in silico method to screen deleterious mutants for the in silico analysis. In the coding regions of TNF-α, NR3C1, and CYP3A5, 14 deleterious mutations were discovered. The protein functional and stability changes in the amino acid between native and mutant energy were identified by analyzing the changes in the hydrogen bonding of these mutants from native, which were all measured using Swiss PDB and PyMOL. F446S and R439K had the highest root-mean-square deviation (RMSD) values among the 14 deleterious mutants. Additionally, the conserved region of amino acid protein interaction was analyzed. This study could aid in the discovery of new detrimental mutations in TNF-α, NR3C1, and CYP3A5, as well as the development of long-term therapy for corticosteroid resistance in several inflammatory diseases. However, more research into the deleterious mutations of the *TNF-α*, *NR3C1*, and *CYP3A5* genes is needed to determine their role in corticosteroid resistance.

## 1. Introduction

Glucocorticoids are pharmacological agents used to treat a variety of illnesses. Glucocorticoids can be endogenous or therapeutic, can affect nearly every tissue in the body, and play an important role in human physiology [1]. However, when patients are treated chronically with glucocorticoids, they develop glucocorticoid resistance or sensitivity, making them more vulnerable to chronic diseases such as asthma, heart disease, and depression [2]. One of the limitations of steroid therapy is the development of resistance to the beneficial effects of glucocorticoids on targeting the tissues [3].

During acute inflammation, macrophages produce many inflammatory cytokines, one of which is TNF-α. It is a proinflammatory cytokine that includes cytotoxic and also immunoregulatory activity. It plays a significant role in B cells, dendritic, and T cells as an immunoregulator and is involved in the execution of the cell death process [4,5]. TNF-α, a potent proinflammatory cytokine, causes an acute form of glucocorticoid resistance. TNF-α has a significant and widespread effect on glucocorticoid transcriptional performance but no effect on nuclear translocation, dimerization, or DNA binding capacity [6]. The human glucocorticoid receptor gene *NR3C1* has nine exons and is located on chromosome 5q31–32. The glucocorticoid receptor polymorphism has an effect on glucocorticoid receptor function, which could be a key factor in glucocorticoid therapy resistance [7]. The *NR3C1* gene, specifically its variants, may influence glucocorticoid treatment towards the target disease. Sensitivity, and treatment outcomes. The exogenous and endogenous molecular mechanisms of glucocorticoids are mainly influenced by intracellular steroid receptors, and NR3C1 encodes them. Glucocorticoid sensitivity is mostly found in the *NR4C1* genes with several SNPs [8]. CYP3A5 belongs to the CYP3A subfamily, and it has a primary role in the metabolism of half of the drugs prescribed globally. Polymorphism in the *CYP3A5* gene severely affects the metabolism and increases the disease condition [9]. The inflammatory transcription gene is downregulated when multiple anti-inflammatory genes are activated or through an independent process synthesis process when the glucocorticoid binds to NR3C1 and CYP3A5 [10,11]. In many cases, mutations or polymorphisms in the *NR3C1* and *CYP3A5* genes may be responsible for glucocorticoid resistance, and treatments are impaired.

Recently, many studies have focused on factors affecting the glucocorticoid function at the molecular level and the genetic variants that played a major role in influencing the mechanism of glucocorticoids. The primary function of glucocorticoids is to suppress the expression of inflammatory genes in a variety of ways via the cytoplasmic receptor interaction in which glucocorticoids interact and inhibit nuclear factor-kappa. (NF-KB) [12]. The resistance of glucocorticoids is caused by the mutation in the amino acids of a gene such as TNF-α, NR3C1, and CYP3A5, while several polymorphisms of this gene are involved in glucocorticoids’ toxicity and response. Thus, due to this condition of glucocorticoid resistance, the anti-inflammatory drugs of glucocorticoids cannot act as a powerful medicine. To understand the pattern of corticosteroid resistance in patients, computational studies are performed on the polymorphisms of the genes such as *TNF-α*, *NR3C1*, and *CYP3A5*.

## 2. Material and Methods

### 2.1. Prediction and Screening of Deleterious SNPs

Using the national center for biotechnology information (NCB1, 1998, United states) and UnitProtKB (Developed by Georgetown university, United states, 2002), the protein sequence and gene of the human TNF-α (Uniport ID: P01375), NR3C1 (Uniport ID: P04150), and CYP3A5 (Uniport ID: P20815) were collected. SNPs are obtained from the dbSNP database (https://www.ncbi.nlm.nih.gov/snp/) (accessed on 22 November 2021) for the *TNF-α*, *NR3C1*, and *CYP3A5* genes. Among them, 403 missense mutations of TNF-α, NR3C1, and CYP3A5 were used for computational analysis.

#### 2.1.1. SIFT and I-Mutant Tools

The normal function of the protein depends on the correct folding of the protein for exhibiting its stability. If not, it may lead to many pathological diseases. Misfolded protein, which leads to loss of their stability, can be studied using the SIFT (https://sift.bii.a-star.edu.sg/) (Introduced in 2001 and supported by bioinformatics institute in Singapore) (accessed on 28 November 2021 ) and I-Mutant tools (https://folding.biofold.org/cgi-bin/i-mutant2.0.cgi) (accessed on 3 December 2021) [13,14]. Sorting intolerance from tolerance was performed on the human nsSNPs available in dbSNP [15]. The phenotypic effect on changes in an amino acid is calculated using this software, and the physiochemical and homology sequences are used for prediction. SIFT predictions are primarily based on amino acid physiochemical properties and sequence homology [16]. The neural network-based web server I-Mutant predicts protein mutations that stabilize or destabilize the protein structure [17].

#### 2.1.2. SNP and GO and PolyPhen-2 Tools

The missense mutation causes a change in amino acids by affecting the structure and function of the protein, which were predicted by the SNP and GO (https://snps-and-go.biocomp.unibo.it/snps-and-go/) (accessed on 10 December 2021) and polyphen-2 (http://genetics.bwh.harvard.edu/pph2/) (accessed on 15 December 2021) tools [18,19]. The SNP and GO server gathered data from a variety of sources, including the protein sequence, SNP local sequence environment, and protein sequence profile. The information from the gene ontology database was analyzed using this server [19]. The values are mentioned after the prediction from zero to a positive number. The value of zero indicates that the SNP has no impact on protein structure, while the positive numbers indicate severe consequences.

### 2.2. Prediction of Site-Directed Mutagenesis

TNF-α (3ALQ) and NR3C1 (1P93) crystal structures are collected from the PDB. The CYP3A5 structure is not available in PDB, and so it was modeled and obtained through SWISS model software (https://swissmodel.expasy.org/) (accessed on 21 December 2021). The amino acid substitution from native to mutant was performed using Swiss PBD viewer 4.1.0 (Developed in 1994 by Nicolas Guex, Swiss institute of bioinformatics, Switzerland. These structures are energy minimized using Swiss PDB viewer 4.1.0 to generate lower energy conformation of a protein structure. During the protein structure modeling, some of the faulty bonded and non-bonded interactions cause structural geometry errors. The energy minimization process is critical for optimizing errors. For the geometry optimization process, the steepest descent algorithm is used [20]. The RMSD value was calculated using PyMOL software 2.5.2 (Created by Warren Lyford Delano and commercialized by schrodinger). The hydrogen bonding variation between the native and mutant was visualized using Swiss PBD viewer 4.1.0 [21,22].

### 2.3. Prediction of Relative Surface Accessibility

Environmental factors affect protein folding through chemicals or temperature when they are exposed, so it is necessary to evaluate the surface accessibility of protein. NetsurfP 2.0 helps to predict the relative surface accessibility of amino acids, and values are calculated as a Z-score from the network reliability score [23].

### 2.4. Analysis of Conserved Amino Acid Residues

The ConSurf server (https://consurf.tau.ac.il) (accessed on 23 December 2021) was used for identifying the position of the amino acid based on evolutionary conservation [24]. The FASTA sequence of the TNF-α, NR3C1, and CYP3A5 proteins is provided to the ConSurf server. The color scheme gives a conservation score from 1–9 (Score 9 means the most conserved amino acid whereas 1 means variable amino acid). ConSurf’s conservation scores represent a relative measure of evolutionary conservation at each target chain sequence site. The lowest score in a protein represents the most conserved position.

### 2.5. String 

The protein–protein interaction studies are important to analyze since the mutated protein continuously affects the other protein during the diseased condition. This helps to study the mechanism of the diseased condition for targeting the source protein and other corresponding proteins. The predicted version of protein–protein interaction information was analyzed using String server 11.5 [25].

## 3. Results and Discussion

*TNF-α*, *NR3C1*, and *CYP3A5* are three genes that play a key role in glucocorticoid resistance and were chosen for computational SNP analysis. Previous computational analysis studies have aided in predicting functional non-synonymous SNPs associated with the *BCL11A* gene [26]. In our current study, we used in silico tools to screen and analyze the SNPs with the deleterious condition and their impact on the TNF-α, NR3C1, and *CYP3A5* genes. Missense variants can also affect the structure of the protein by affecting the interaction, stability, and solubility of the protein. To evaluate the effect of a missense mutation on protein structure and function, the SNPs are mapped into the protein structure and validated through in silico [27,28,29]. The human gene of *TNF-α*, *NR3C1*, and *CYP3A5* contains a total of 1119 missense mutations in the NCBI dbSNP database. In this study, randomly, 403 missense mutations of TNF-α (118), NR3C1 (141), and CYP3A5 (144) were retrieved from the dbSNP database. Finding SNPs responsible for specific characteristics using molecular techniques looks to be costly. As a result, in silico techniques can help in genetic association studies and acquire a better understanding of the parent protein’s functional and structural characteristics [30]. The selected nsSNPs were tested by SIFT, I-Mutant, polyphen-2, and SNP and GO tools to see if they changed protein stability due to mutation and deleterious. Previously, it was reported that many deleterious SNPs from *BCL11B*, *VDR*, and *CYP24A1* gene are identified using these tools [1,26]

SIFT prediction helps to analyze the function of the protein in case of a change in amino acid and allows for the prioritization of substitutions for further investigation [31]. It speculates on whether or not the substitution is deleterious or tolerated. SIFT values of less than 0.05 are harmful, while values higher than 0.05 are harmless. The selected missense mutations of SNPs were tested by I-Mutant to investigate the change in protein stability due to mutation [32]. PolyPhen-2 is used for studying protein function and structure through information obtained from phylogenetic, structural, and sequence analysis. Deleterious (1.0) and tolerated (0.0) SNPs are identified based on the score value [18]. SNPs and GO can predict mutation-induced disease using protein sequence and functional protein annotation. A probability score greater than 0.5 indicates that the mutation has a disease-related effect on the parent protein function [19]. The findings from SNPs of TNF-α, NR3C1, and CYP3A5 indicate that among the 403 missense mutation, 14 SNPs were predicted from various tools such as SNP, I-Mutant, SNP and GO, polyphen-2 and are presented in Table 1. From the outcomes of these four servers, it was concluded that in TNF-α, there were five deleterious SNPs with rsIDs of rs11574936 (I194N), rs140654183 (T181N), rs190788828 (K87T), rs369510319 (R158H), and rs566451995 (A172V). Then, from NR3C1, there were five deleterious nsSNPs with rsIDs of rs104893913 (R477H), rs104893909 (I559N), rs104893914 (G679S), rs121909726 (L753F), rs6190 (R23T), rs6189 (E22D), and rs104893911 (V571A). Further, CYP3A5 had four deleterious nsSNPs with rsIDS of rs41279854 (F446S), rs13220949 (R439K), rs72552791 (Y53C), and rs140521496 (P416S). It was previously reported that mutations and small deletions in the *NR3C1* gene were the cause of generalized glucocorticoid resistance syndrome [33].

**Figure 1:**
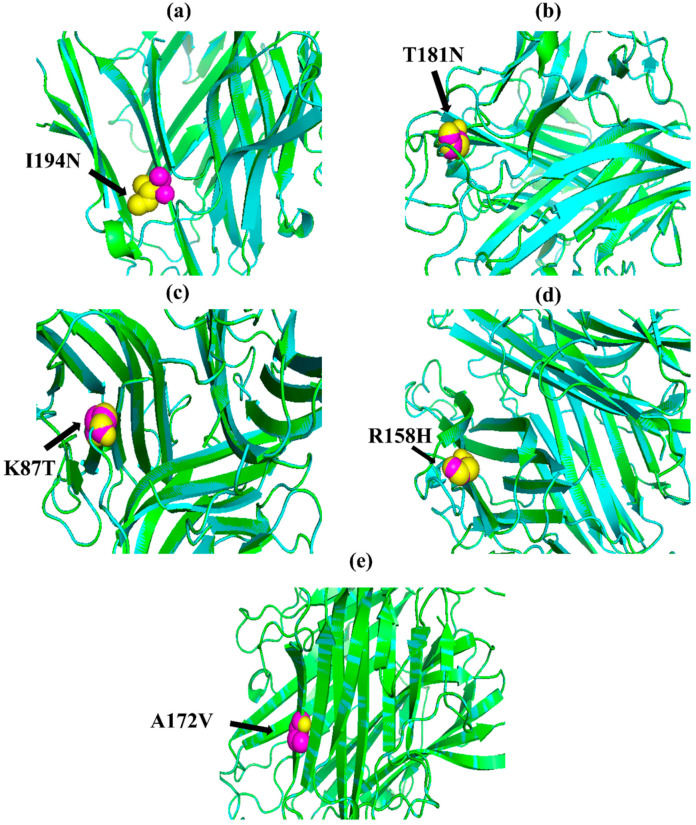
PyMOL was used to visualize 3D structure comparison modeling of the TNF-α protein. (**a**) At position 194, the native amino acid isoleucine (yellow) with the mutant amino acid asparagine (purple). (**b**) At position 181, the native amino acid threonine (yellow) with the mutant amino acid asparagine (purple). (**c**) The native amino acid lysine (yellow) with the mutant amino acid threonine (purple) at position 87. (**d**) The native amino acid arginine (yellow) is with the mutant amino acid histidine (purple) at position 158. (**e**) At position 172, the native amino acid alanine (yellow) with the mutant amino acid valine (purple).

**Figure 2:**
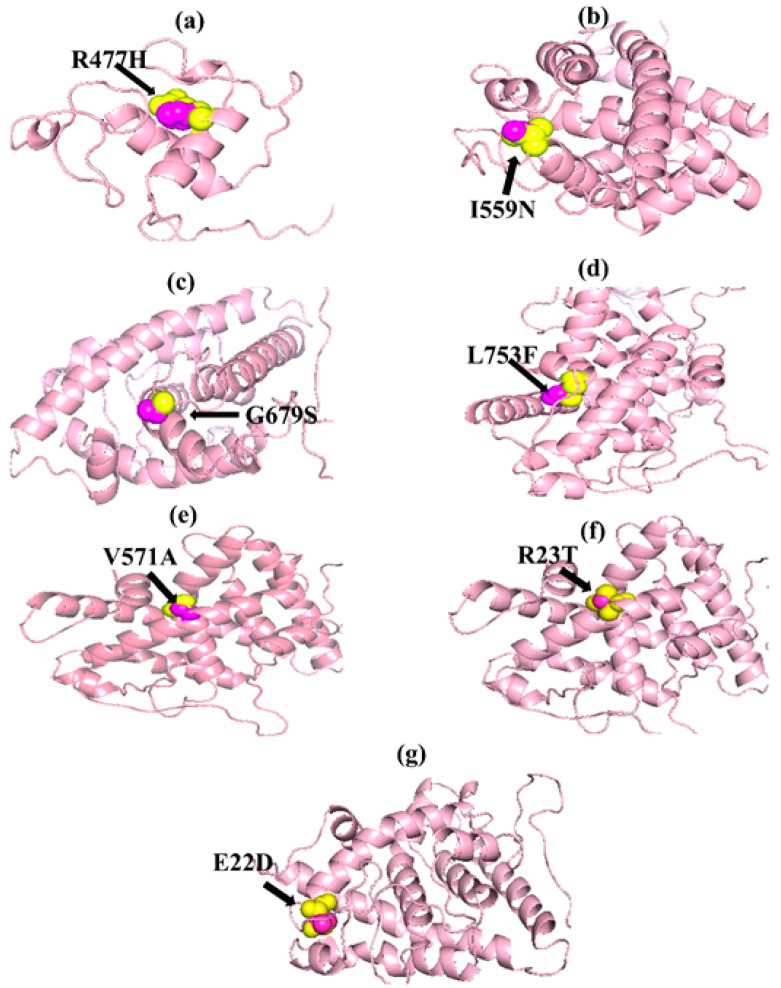
PyMOL was used to visualize 3D structure comparison modeling of the NR3C1 protein. (**a**) The native amino acid arginine (yellow) with the mutant amino acid histidine (purple) at position 477. (**b**) At position 559, the native amino acid isoleucine (yellow) with the mutant amino acid asparagine (purple). (**c**) A sphere-shaped native amino acid glycine (yellow) with a mutant amino acid serine (purple) at position 679. (**d**) At position 753, the native amino acid leucine (yellow) with the mutant amino acid phenylalanine (purple). (**e**) At position 571, the native amino acid valine (yellow) with the mutant amino acid alanine (purple). (**f**) At position 23, the native amino acid arginine (yellow) with the mutant amino acid threonine (purple). (**g**) At position 22, the native amino acid glutamic acid (yellow) with the mutant amino acid aspartic acid (purple).

**Figure 3:**
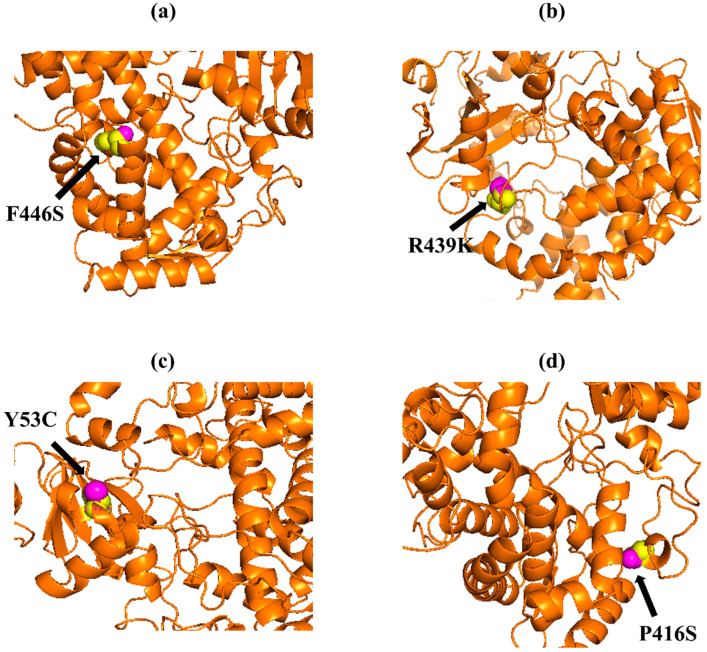
PyMOL was used to visualize 3D structure comparison modeling of the CYP3A5 protein. (**a**) A sphere-shaped native amino acid phenylalanine (yellow) with a mutant amino acid serine (purple) at position 446. (**b**) The native amino acid arginine (yellow) with the mutant amino acid lysine (purple) at position 439. (**c**) A sphere-shaped native amino acid tyrosine (yellow) with a mutant amino acid cysteine (purple) at position 53. (**d**) The native amino acid proline (yellow) with the mutant amino acid serine (purple) at position 416.

**Figure 4:**
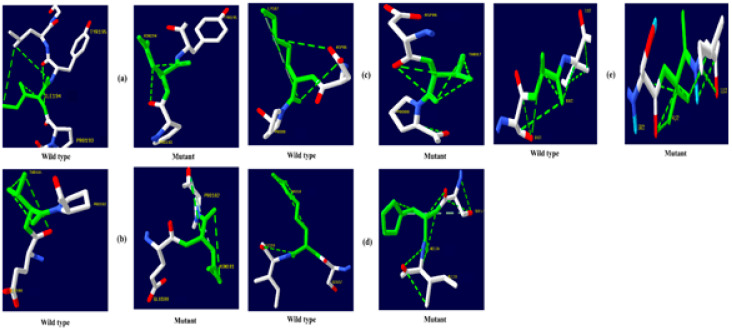
TNF-α hydrogen bonding results (**a**) I194N, (**b**) T181N, (**c**) K87T, (**d**) R158H, and (**e**) A172V.

**Figure 5:**
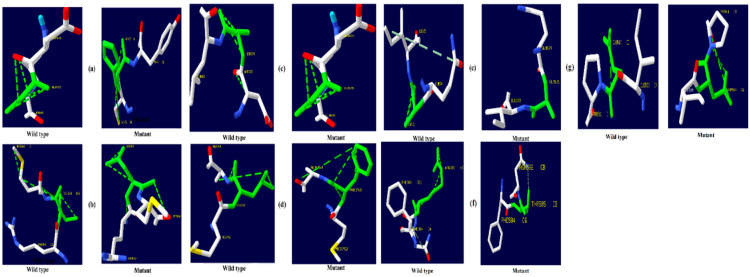
NR3C1 protein hydrogen bonding results (**a**) R477H, (**b**) I559N, (**c**) G679S, (**d**) L753F, (**e**) V571A, (**f**) R23T, and (**g**) E22D.

**Figure 6:**
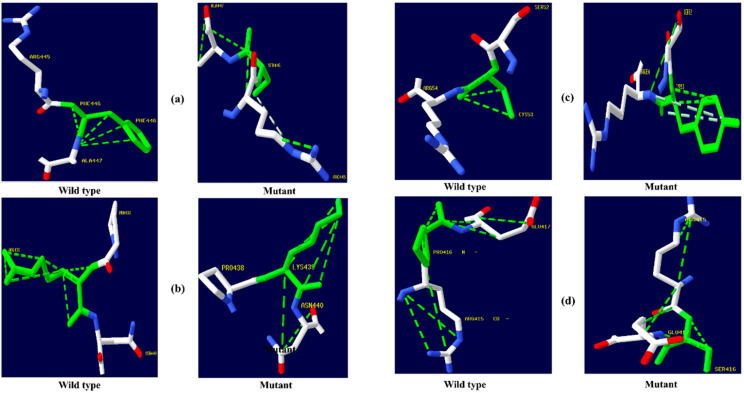
CYP3A5 protein hydrogen bonding results (**a**) F446S, (**b**) R439K, (**c**) Y53C, and (**d**) P416S.

The native amino acid of the TNF-α, NR3C1, and CYP3A5 proteins was changed to the mutant amino acid using the Swiss PDB for comparative modeling. The modeling of the 3D structure of the protein with mutant and native residues helps to visualize the changes in the amino acids and their structural modification of a protein. The superimposed structure with native to mutant amino acid (Figure 1, Figure 2 and Figure 3) was created with PyMOL. The structure analysis of TNF-α (PDB ID: 3ALQ) was performed using Swiss PDB. The software maps the SNPs by replacing the amino acid with its mutant and testing for various properties. The RMSD is a commonly used metric for comparing values predicted by a model or estimator to values observed. The RMSD value can be used to measure the backbone distance between the proteins in superimposed structures. The values of RMSD are calculated based on the square root of the averaged square error. The translation and rotation of one structure with respect to the other is a common way to compare the structures of biomolecules or solid bodies to minimize RMSD [13,34]. The greater the RMSD will be when it is a loop, and these scores are measured by comparing the RMSD between the native and mutant [35]. The RMSD calculated by PyMOL revealed that the amino acid changes I194N, T181N, K87T, R158H, and A172V in the TNF-α protein have scores of 0.04, 0.02, 0.03, 0.03, and 0.03 (Table 2). Similarly, RMSD values for the NR3C1 and CYP3A5 proteins are mentioned in Table 3 and Table 4. According to the previous report, RMSD analysis revealed a difference in values between mutant and native on the *NR3C1* gene, which causes glucocorticoid resistance [36]. The structure and function of protein rely heavily on hydrogen bonds and other nonbonding interactions [37]. Hence, the Swiss PDB viewer was used to examine hydrogen bonding patterns in both native and mutant structures of both proteins. A change in the position of the hydrogen bond was observed in the proteins TNF-α, NR3C1, and CYP3A5. These findings suggest that these mutations may significantly impact the protein’s structure, function, and stability compared to the native form. Figure 4, Figure 5 and Figure 6 represent the changes in the hydrogen bond. Previous research has shown that a missense mutation in the human glucocorticoid receptor resulted in glucocorticoid resistance by disrupting the hydrogen bond [38]. The structure and functions of proteins are influenced by solvent accessibility and hydrophobicity [39]. Polar side chains in proteins are more likely to be exposed to the solvent, whereas hydrophobic residues are more likely to be buried deep within the protein, away from the solvent. Protein stability improves as the area of water-accessible hydrophobic surface decreases [40,41]. The above-mentioned variants of TNF-α, NR3C1, and CYP3A5 were evaluated for solvent accessibility and stability using the NetsurfP server 2.0. The obtained results are mentioned in Table 5, Table 6 and Table 7. Mutations in buried sites are more likely to disrupt the protein structure. After further investigation, it was discovered that the mutant type relative solvent accessibility (RSA), and accessible surface area (ASA) values of TNF, NR3C1, and CYP3A5 have changed compared to the native type. The same difference was seen in the Z fit score, indicating that SNP has an impact on protein structure changes. It was previously shown that glucocorticoid resistance is caused by protein structural alteration in the glucocorticoid receptor [42].

**Figure 7:**
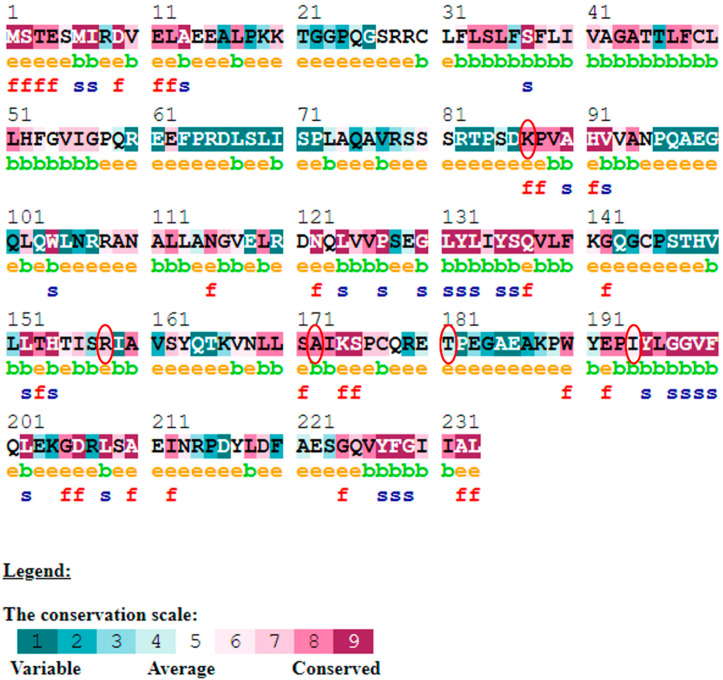
ConSurf server predicted conserved amino acids in TNF-protein. Based on the conservation scale, the amnio acids are ranked as variable, average, and conserved.

**Figure 8:**
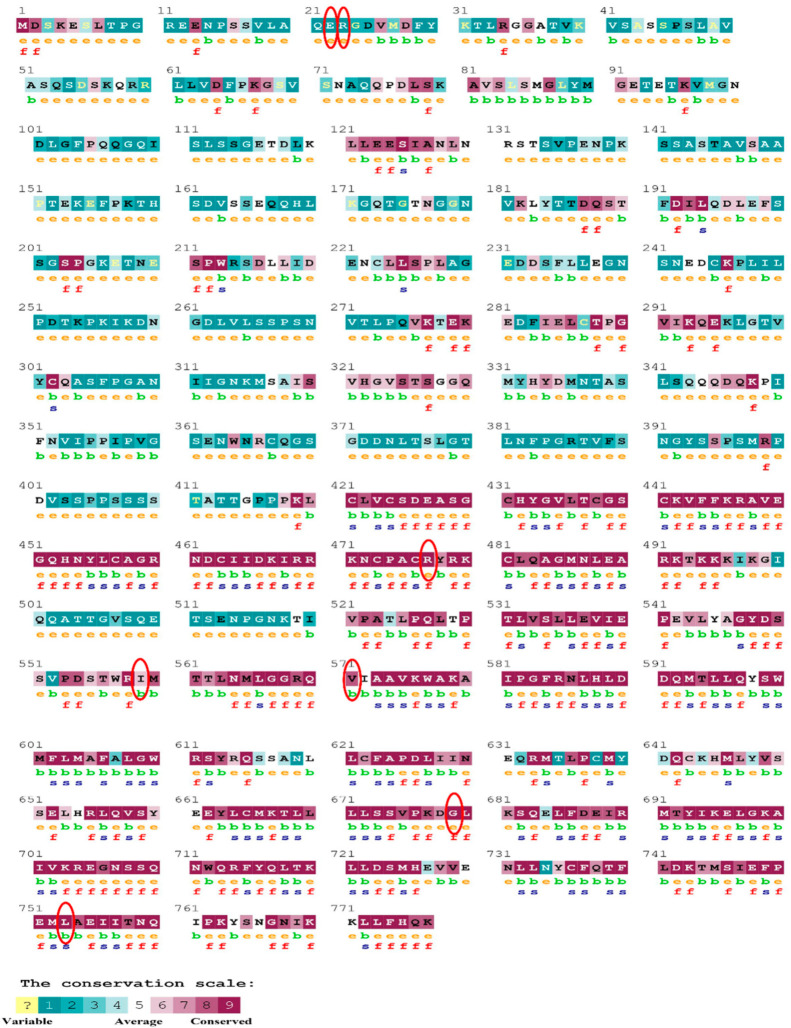
The ConSurf server predicted conserved amino acids in the NR3C1 protein. Based on the conservation scale, the amnio acids are ranked as variable, average, and conserved.

**Figure 9:**
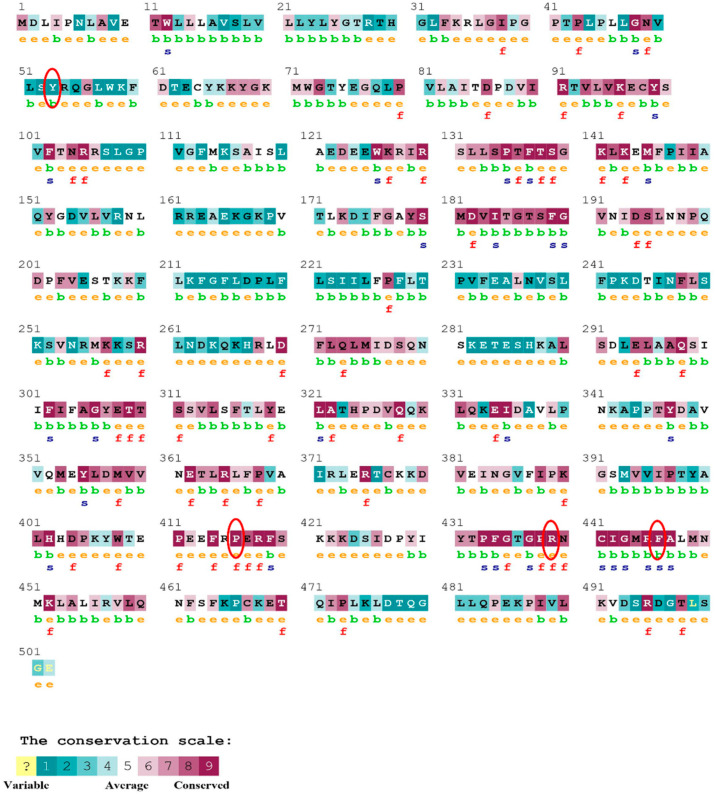
The ConSurf server predicted conserved amino acids in the CYP3A5 protein. Based on the conservation scale, the amnio acids are ranked as variable, average, and conserved.

**Figure 10:**
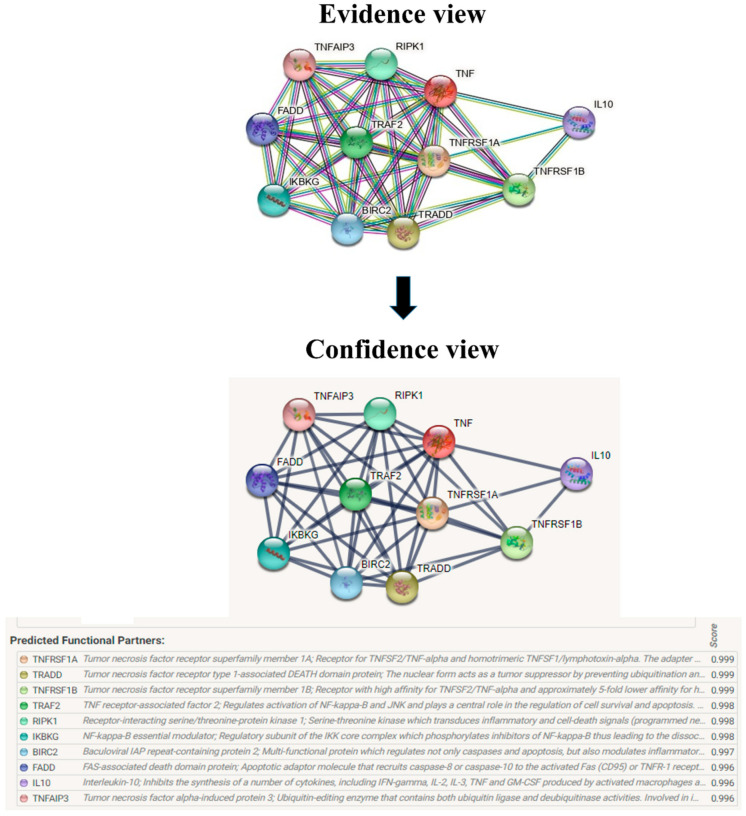
Overview of TNF-α network construction using String server 11.5. The evidence view and confidence view are given.

**Figure 11:**
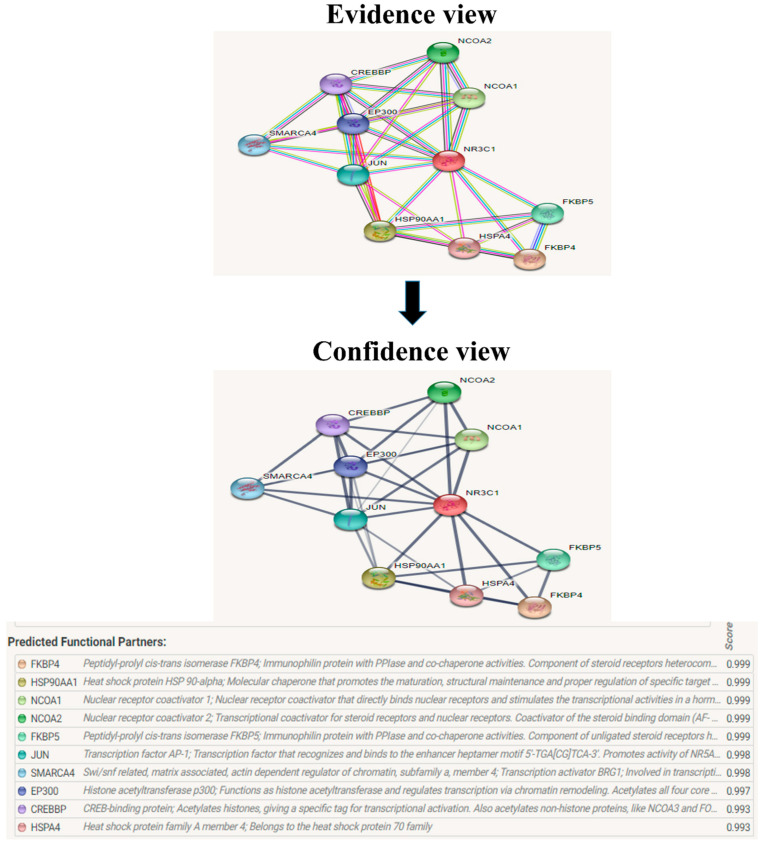
The protein NR3C1 interacts with a total of 10 other proteins. The evidence view and confidence view are given, while the dark blue lines denoting a strong correlation.

**Figure 12:**
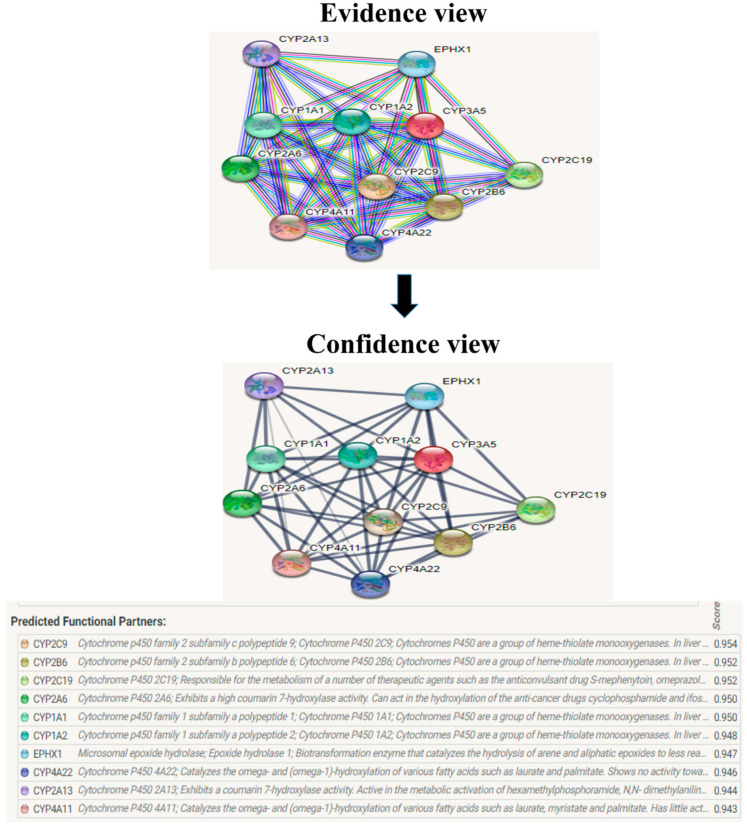
CYP3A5 interacts with a total of 10 different proteins. The evidence view and confidence view are given, while dark blue lines indicating a strong link.

The evolutionary rate is calculated in ConSurf based on the evolutionary relationship between the protein and its homologs and the amino acid similarity as reflected in the substitutions matrix. The residues R158 and A172 are conserved and exposed with a score of 8 in the TNF-α protein (Figure 7). The R477, G679, and L753 residues in the NR3C1 protein are highly conserved and exposed, with a score value of 9 (Figure 8). The residues R439 and P416 in the CYP3A5 protein, on the other hand, are highly conserved, whereas the other residues F446 and Y53 are variable (Figure 9) [24]. Further, the interaction of the TNF-α, NR3C1, and CYP3A5 proteins with other corresponding proteins which may affect the signaling pathway was studied using the STRING database. Both confidence and evidence views have been shown in Figure 10, Figure 11 and Figure 12. It has been observed that there is a strong functional association of TNF-α protein with IL10, RIPK1, TNFRSF1B, TRADD, BIRC2, IKBKG, FADD, TNFAIP3, TRAF2, and TNFRSF1A. For the interaction of NR3C1 protein, it was found by NCOA2, NCOA1, FKBP5, FKBP4, HSPA4, HSP90AA1, JUN, CREBBP, and SMARCA4. The CYP3A5 protein was found to interact with EPHX1, CYP2C19, CYP2B6, CYP4A22, CYP4A11, CYP2A6, CYP1A1, CYP2A13, CYP1A2, and CYP2B6. The results showed that mutation in the residues of these proteins showed that changes in amino acids could interfere with other associated proteins. We sought to anticipate SNPs that can change protein expression and function in three interconnected genes in this work (*TNF-α*, *NR3C1*, and *CYP3A5*). Mutations in these genes have been linked to a variety of disorders. Interestingly, our in silico studies reveal the detrimental nature of these SNPs. As a result, our data obscure the possibility that these mutations alter gene expression and protein structure. As a result, alterations in amino acids in a specific location may be linked to glucocorticoid resistance. As a result, our research could help refine SNP prediction by identifying SNPs with a high potential for complexity.

## 4. Conclusions

According to the findings, mutants of TNF-α, NR3C1, and CYP3A5 are highly deleterious, and their presence can result in protein under-expression. Similarly, mutants of TNF-α, NR3C1, and CYP3A5 have been discovered to affect protein structure and stability, potentially leading to protein dysfunction. As a result of these mutations, glucocorticoid resistance may develop. The comparative in silico analysis of these gene variants showed a potential application for large-scale research. The current study will also aid experimental geneticists in their large-scale SNP analysis and assist in finding functional variation from the perspectives of structure, expression, evolution, physiochemical property, and phenotypes. This bioinformatics research will need to be looked at further in our future human clinical trials to see if the in silico study can be linked to the clinical trial. However, since TNF-α, NR3C1, and CYP3A5 are involved in a key mechanism of glucocorticoid resistance, their nsSNPs can aid in diagnosing and treating the condition.

## Figures and Tables

**Table 1:** Prediction of the effect of SNPs by using SIFT, I-Mutant, SNP&GO, and PolyPhen-2 server.

Gene	Uniprot ID	SNP ID	Amino Acid Changes	SIFT	I-Mutant	SNP&GO	Polyphen-2
*TNF-α*	P01375	rs11574936	I194N	Damaging	Decrease	Disease	Disease
		rs140654183	T181N	Damaging	Decrease	Disease	Disease
		rs190788828	K87T	Damaging	Decrease	Disease	Disease
		rs369510319	R158H	Damaging	Decrease	Disease	Disease
		rs566451995	A172V	Damaging	Decrease	Disease	Disease
*NR3C1*	P04150	rs104893913	R477H	Damaging	Decrease	Disease	Disease
		rs104893909	I559N	Damaging	Decrease	Disease	Disease
		rs104893914	G679S	Damaging	Decrease	Disease	Disease
		rs121909726	L753F	Damaging	Decrease	Disease	Disease
		rs6190	R23T	Damaging	Decrease	Disease	Disease
		rs6189	E22D	Damaging	Decrease	Disease	Disease
		rs104893911	V571A	Damaging	Decrease	Disease	Disease
*CYP3A5*	P20815	rs41279854	F446S	Damaging	Decrease	Disease	Disease
		rs13220949	R439K	Damaging	Decrease	Disease	Disease
		rs72552791	Y53C	Damaging	Decrease	Disease	Disease
		rs140521496	P416S	Damaging	Decrease	Disease	Disease

**Table 2:** RMSD value of TNF-α protein.

Gene	Uniprot ID	SNP ID	Amino Acid Change	RMSD(Å)
*TNF-α*	P01375	rs11574936	I194N	0.04
		rs140654183	T181N	0.02
		rs190788828	K87T	0.03
		rs369510319	R158H	0.03
		rs566451995	A172V	0.03

**Table 3:** RMSD value of NR3C1 protein.

Gene	Uniprot ID	SNP ID	Amino Acid Change	RMSD(Å)
*NR3C1*	P04150	rs104893913	R477H	1.7
		rs104893909	I559N	1.9
		rs104893914	G679S	1.8
		rs121909726	L753F	2.3
		rs6190	R23T	1.6
		rs6189	E22D	1.7
		rs104893911	V571A	2.9

**Table 4:** RMSD value of CYP3A5 protein.

Gene	Uniprot ID	SNP ID	Amino Acid Change	RMSD(Å)
		rs41279854	F446S	5.3
*CYP3A5*	P20815	rs13220949	R439K	5.3
		rs72552791	Y53C	4.9
		rs140521496	P416S	4.6
		rs104893911	V571A	2.9

**Table 5:** Prediction of relative surface accessibility of TNF-α through NetsurfP.

Gene	Mutation	NetsurfP			
		Class	RSA	ASA	Z Fit Score
*TNF-α*	I194N	Buried	0.067	12.45	−0.365
		Exposed	0.462	67.578	−1.221
	T181N	Exposed	0.501	69.489	−1.623
		Exposed	0.565	82.643	−0.709
	K87T	Exposed	0.349	71.789	0.357
		Exposed	0.368	51.055	0.588
	R158H	Buried	0.189	43.166	0.513
		Buried	0.161	29.213	−0.081
	A172V	Exposed	0.469	51.717	−1.707
		Exposed	0.432	66.383	−1.871

**Table 6:** Prediction of relative surface accessibility of NR3C1 through NetsurfP.

Gene	Mutation	NetsurfP			
		Class	RSA	ASA	Z Fit Score
*NR3C1*	R477H	Buried	0.155	35.449	−0.288
		Buried	0.178	32.324	−0.183
	I559N	Exposed	0.378	70.004	−1.158
		Exposed	0.341	49.878	−0.937
	G679S	Exposed	0.389	30.591	−1.456
		Exposed	0.389	45.626	−1.597
	L753F	Buried	0.028	5.145	0.956
		Buried	0.029	5.84	0.777
	R23T	Exposed	0.396	90.661	−0.661
		Exposed	0.427	59.225	−0.744
	E22D	Exposed	0.446	77.829	−1.100
		Exposed	0.580	83.549	−1.691
	V571A	Exposed	0.100	15.324	−0.021
		Exposed	0.078	8.629	−0.142

**Table 7:** Prediction of relative surface accessibility of CYP3A5 through NetsurfP.

Gene	Mutation	NetsurfP			
		Class	RSA	ASA	Z Fit Score
*CYP3A5*	F446S	Buried	0.076	15.293	−0.242
		Buried	0.078	9.165	−0.177
	R439K	Buried	0.162	37.19	−0.931
		Buried	0.174	35.854	−1.07
	Y53C	Buried	0.107	22.887	−1.153
		Buried	0.095	13.38	−0.499
	P416S	Buried	0.122	17.34	−0.13
		Buried	0.127	14.873	−0.116

## Data Availability

Data presented are available on request by the corresponding authors.
